# Successful Percutaneous Coronary Intervention (PCI) of a Heavily Calcified Left Main Artery Using Shockwave Intravascular Lithotripsy and Intra-aortic Balloon Pump (IABP) Support in an Octogenarian Acute Coronary Syndrome Patient Deemed High Risk

**DOI:** 10.7759/cureus.83585

**Published:** 2025-05-06

**Authors:** Hassan Elzain, Nader Alasousi, Mohamed Adel Mostafa, Khalidenbalwaleed Ali, Anas Babiker

**Affiliations:** 1 Cardiology, Ministry Of Health-Kuwait, Kuwait City, KWT; 2 Cardiology, Royal Care International Hospital, Khartoum, SDN

**Keywords:** complex pci, coronary artery angiography, coronary artery bypass grafting(cabg), coronary artery intervention, interventional cardiologist, left main stenosis, shockwave intravascular lithotripsy

## Abstract

Managing heavily calcified left main (LM) disease in elderly patients presenting with acute coronary syndrome (ACS) presents a significant clinical challenge, particularly when surgical revascularization is considered high risk. Intravascular lithotripsy (IVL) has emerged as a promising technique for calcium modification in high-risk coronary interventions. We report the case of an 81-year-old woman with a history of diabetes mellitus, hypertension, stage 4 chronic kidney disease, and chronic obstructive pulmonary disease who presented with anterior acute coronary syndrome. Coronary angiography revealed heavily calcified LM disease with triple vessel involvement. The cardiothoracic surgery team assessed her as high risk for surgical revascularization due to her advanced age and multiple comorbidities. Echocardiography showed regional wall motion abnormalities with moderately reduced ejection fraction (40%), and high-sensitivity troponin I was markedly elevated (>24,000 ng/L). Percutaneous coronary intervention (PCI) was performed with intra-aortic balloon pump (IABP) support. Shockwave IVL was used to prepare the calcified LM lesion, followed by successful stenting with restoration of Thrombolysis in Myocardial Infarction (TIMI) III flow. The patient had an uneventful recovery and was discharged on optimal medical therapy. This case highlights the feasibility and safety of combining IVL and IABP support in treating heavily calcified left main lesions in elderly ACS patients deemed high risk for surgical revascularization. It underscores the importance of individualized decision-making and the role of advanced calcium-modification technologies in high-risk coronary interventions.

## Introduction

Left main coronary artery (LMCA) disease represents one of the most critical manifestations of coronary artery disease, owing to its anatomical importance in supplying a significant portion of the myocardium. It accounts for approximately 4% to 6% of coronary artery lesions identified during angiography and carries substantial morbidity and mortality risk due to the potential for extensive myocardial ischemia [1,2]. Historically, coronary artery bypass grafting (CABG) has been the gold standard for revascularization in LMCA disease, particularly when complicated by multivessel disease, as it demonstrates superior outcomes in terms of survival and reduced need for repeat intervention [3,4]. However, advancements in percutaneous coronary intervention (PCI), including the use of modern drug-eluting stents and adjunctive tools such as intravascular lithotripsy (IVL), have allowed for safer and more effective management of heavily calcified coronary lesions, even in high-risk patients traditionally deemed unsuitable for PCI [5]. We present a case of an elderly patient with heavily calcified left main coronary artery disease complicated by multiple comorbidities who was successfully treated with PCI utilizing IVL and intra-aortic balloon pump (IABP) support, highlighting the evolving role of tailored percutaneous interventions in high-risk populations.

## Case presentation

An 81-year-old woman with a history of type 2 diabetes mellitus, hypertension, stage 4 chronic kidney disease (CKD), and chronic obstructive pulmonary disease (COPD) presented to the emergency department with acute central chest pain that began two hours prior to arrival. The pain was pressure-like, radiated to the left arm, and was associated with shortness of breath and diaphoresis. She had no prior history of ischemic heart disease or coronary interventions.

**Figure 1:**
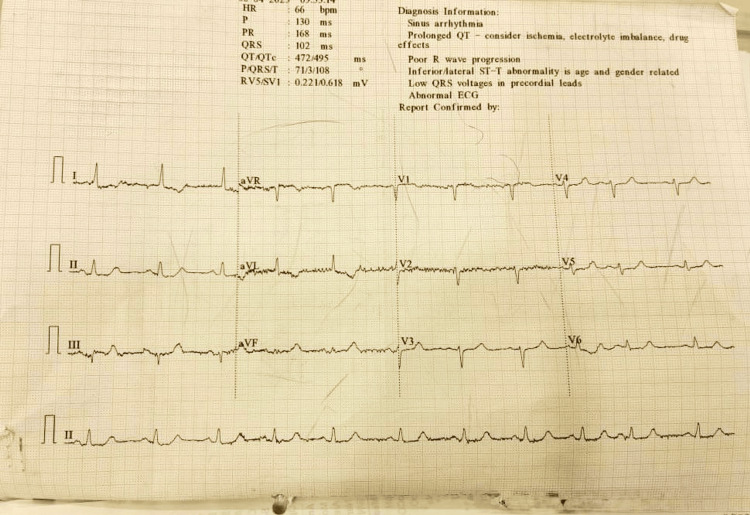
ECG The electrocardiogram shows ST-segment elevation in lead aVR with widespread ST-segment depression in the lateral and inferior leads, consistent with left main or multivessel ischemia.

On initial assessment, the patient was hemodynamically stable with a blood pressure of 110/60 mmHg, heart rate of 66 beats per minute, and oxygen saturation of 94% on room air. The initial electrocardiogram showed ST elevation in lead aVR with widespread ST depression in the lateral and inferior leads, consistent with a high-risk ischemia (Figure 1).

Laboratory investigations revealed a markedly elevated high-sensitivity troponin I (>24,000 ng/L), hemoglobin of 9.8 g/dL, and serum creatinine of 191 µmol/L (estimated glomerular filtration rate (eGFR) 21 mL/min/1.73 m²), consistent with her baseline renal function (Table 1).

**Table 1:** Laboratory Investigations eGFR: estimated glomerular filtration rate, CRP: C-reactive protein, LDL: low-density lipoprotein, HDL: high-density lipoprotein, HbA1C: hemoglobin A1C, BNP: B-type natriuretic peptide

Test	Result	Reference Range	Units
Hemoglobin	9.8 g/dL	12–16	g/dL
WBC	4.2 ×10⁹/L	4.0–11.0	×10⁹/L
Platelet Count	237 ×10⁹/L	150–410	×10⁹/L
Creatinine	191 µmol/L	45–90	µmol/L
eGFR	21 mL/min/1.73m²	>60	mL/min/1.73m²
Urea	24.8 mmol/L	2.8–7.2	mmol/L
CRP	23.2 mg/L	<5	mg/L
Troponin I	>24,000 ng/L	<8	ng/L
Na+	142 mmol/L	135–145	mmol/L
K+	4.9 mmol/L	3.5–5.0	mmol/L
Cl−	102 mmol/L	95–105	mmol/L
Ca2+	2.19 mmol/L	2.2–2.65	mmol/L
Phosphate	1.93 mmol/L	0.81–1.45	mmol/L
Mg2+	0.91 mmol/L	0.71–1.03	mmol/L
Albumin	36 g/L	35–52	g/L
LDL Chol	2.9 mmol/L	<3.4	mmol/L
HDL Chol	1.34 mmol/L	>1.0	mmol/L
Triglycerides	1.75 mmol/L	<1.7	mmol/L
HbA1c	10.1%	4–6%	%
BNP	13,594 pg/mL	<300	pg/mL

Transthoracic echocardiography showed concentric left ventricular remodeling with regional wall motion abnormalities involving the apical cap, apical septum, inferoseptum, and anteroseptum. The left ventricular ejection fraction was moderately reduced at approximately 40% (Videos 1, 2)

**Video 1:** Echocardiography Parasternal short axis view at the level of papillary muscles showing the regional wall motion abnormalities

**Video 2:** Echocardiography Transthoracic echocardiography showed concentric left ventricular remodeling with regional wall motion abnormalities involving the apical cap, apical septum, inferoseptum, and anteroseptum. The left ventricular ejection fraction was moderately reduced at approximately 40%.

In detailed echocardiography, the mitral valve appeared thickened with restricted leaflet motion, consistent with mild degenerative mitral stenosis, and there was associated mild-to-moderate mitral regurgitation (Video 3).

**Video 3:** Echocardiography Four chambers view with color flow Doppler showing mitral regurgitation

The tricuspid regurgitation velocity was elevated, with an estimated right ventricular systolic pressure of approximately 70 mmHg. The inferior vena cava was dilated at 2.7 cm and showed no inspiratory collapse.

Diagnostic coronary angiography revealed significant triple vessel disease with a severely calcified and critically stenotic left main coronary artery (90% from ostium to proximal segment). The proximal left anterior descending artery exhibited 60-70% stenosis with heavy calcification, and the proximal left circumflex artery showed 50-70% calcified stenosis. The right coronary artery was dominant and demonstrated proximal stenosis of approximately 80% (Video 4).

**Video 4:** Coronary Angiography Diagnostic coronary angiography revealed significant triple vessel disease with a severely calcified and critically stenotic left main coronary artery (90% from ostium to proximal segment). The proximal left anterior descending artery exhibited 60–70% stenosis with heavy calcification, and the proximal left circumflex artery showed 50–70% calcified stenosis. The right coronary artery was dominant and demonstrated proximal stenosis of approximately 80%.

Due to the critical nature of the findings and the patient’s high-risk profile, an IABP was inserted for hemodynamic support, and the case was referred for urgent surgical evaluation.

The case was discussed in a multidisciplinary meeting involving the interventional cardiology and cardiothoracic surgery teams. Given the critical left main disease with multivessel involvement and the hemodynamic risk, surgical revascularization via CABG was initially considered. However, the cardiothoracic team deemed the patient high-risk due to multiple factors, including her advanced age (81 years), stage 4 chronic kidney disease (eGFR 21 mL/min/1.73 m²), chronic obstructive pulmonary disease, moderate pulmonary hypertension (right ventricular systolic pressure (RVSP) ~70 mmHg), and anemia. After detailed discussion of the risks and treatment options, the patient opted to proceed with PCI.

Percutaneous coronary intervention was performed with IABP support. Given the severe calcification of the left main lesion, we initially performed pre-dilatation using a 2.5 × 15 mm non-compliant balloon. Lesion preparation was then achieved using a shockwave intravascular lithotripsy balloon (3.5 × 12 mm), delivering multiple cycles of pulse therapy (Video 5).

**Video 5:** Balloon Dilatation of the left main Ballooning of the left main.

Then a drug-eluting stent measuring 3.5 × 15 mm was deployed from the left main into the proximal left anterior descending artery (LAD) with good expansion and full lesion coverage, followed by post-dilatation using a 4.5 × 15 mm non-compliant balloon to optimize stent expansion.

Following stenting of the left main, the ostium of the left circumflex artery appeared pinched. The LCx was wired and treated with balloon dilatation using a 2.5 × 15 mm non-compliant balloon, with no further stenting required. Finally, the proximal right coronary artery was treated with a 3.5 × 20 mm drug-eluting stent. The patient remained hemodynamically stable throughout the procedure, which was completed without complications. Final angiography demonstrated Thrombolysis in Myocardial Infarction (TIMI) III flow in all treated vessels with no residual stenosis (Video 6).

**Video 6:** Final Result Good result with TIMI3 Flow in all vessels

Efforts were made to minimize contrast volume due to the patient’s underlying chronic kidney disease; a total of 150 mL of contrast was used throughout the procedure. The IABP was removed six hours after the procedure. On the second day, the patient developed oliguria with a mild rise in serum creatinine above baseline. She was managed conservatively with close monitoring and supportive care. Her renal function gradually improved, returning to baseline by day five. She was discharged in stable condition. At follow-up one week later, she was asymptomatic and her renal function remained at baseline.

## Discussion

Management of significant LMCA disease, particularly when associated with multivessel coronary artery disease, remains challenging despite advances in coronary interventions. While PCI with drug-eluting stents has become increasingly feasible in treating isolated LMCA lesions with low anatomical complexity [5], surgical revascularization through CABG continues to be the preferred approach for complex LMCA and multivessel disease due to its long-term survival benefits and lower rates of repeat revascularization [3,4]. Data from large randomized controlled trials, such as SYNTAX and EXCEL, have reinforced the superiority of CABG over PCI in patients with higher anatomical complexity or additional vessel involvement [3,4].

In our case, the presence of extensive calcification and multivessel involvement significantly increased procedural complexity. Furthermore, advanced age and multiple comorbidities, including chronic kidney disease, chronic obstructive pulmonary disease, and pulmonary hypertension, made surgical intervention particularly high-risk, leading the surgical team to recommend against CABG. After careful consideration and detailed discussion of potential risks and benefits with the patient, a decision was made to proceed with PCI utilizing adjunctive techniques, including IABP support and shockwave IVL.

The presence of significant comorbidities in elderly patients poses substantial challenges for surgical revascularization. Advanced age alone increases the operative risk for CABG, and this risk is further compounded by conditions such as CKD, COPD, pulmonary hypertension, and anemia [6,7]. Patients with advanced CKD, particularly those with stage 4 or worse, are at higher risk of postoperative acute kidney injury, which significantly increases morbidity and mortality following cardiac surgery [8]. Similarly, COPD and pulmonary hypertension independently elevate the perioperative risk, contributing to prolonged hospitalization, increased mechanical ventilation duration, and heightened mortality risk [9].

In our case, after comprehensive evaluation by the multidisciplinary Heart Team, the cardiothoracic surgery team classified the patient as high-risk due to her advanced age, severe CKD, moderate pulmonary hypertension, COPD, and anemia. Consequently, the surgical team recommended against CABG as the primary revascularization strategy. Following detailed communication about the potential risks and benefits of alternative treatment options, the patient expressed her preference for PCI, emphasizing the importance of patient-centered shared decision-making in complex clinical scenarios [10].

The use of IABP support during high-risk PCI remains a common strategy to enhance procedural safety, particularly in patients with severe left ventricular dysfunction, complex coronary lesions, or hemodynamic instability. The IABP works by reducing afterload, improving coronary perfusion, and augmenting systemic perfusion through synchronized balloon inflation and deflation during the cardiac cycle [11,12]. Although routine use of IABP in all high-risk PCI procedures remains controversial, selective use in patients deemed to have significant procedural or clinical risks is generally supported by current guidelines and expert consensus [13].

Multiple studies have demonstrated that elective use of IABP during PCI in patients with severely reduced left ventricular function, significant left main disease, or hemodynamic compromise improves procedural hemodynamics and may enhance clinical outcomes, including reduced rates of procedural complications and improved short-term survival [12,13]. Conversely, some randomized studies such as the BCIS-1 trial showed no clear mortality benefit of routine IABP use in stable but high-risk elective PCI patients, reinforcing the concept of selective rather than routine application [14].

In our patient, given the presence of extensive calcification in the critical left main lesion, significant multivessel coronary disease, and multiple high-risk comorbidities, the Heart Team opted for prophylactic IABP support. The patient remained stable hemodynamically throughout the procedure without complications, underscoring the potential benefits of individualized decision-making in using hemodynamic support devices in selected high-risk cases.

PCI for severely calcified coronary lesions remains technically challenging due to difficulties in achieving optimal stent expansion, increasing the risk of procedural complications and subsequent restenosis or thrombosis. In recent years, Shockwave IVL has emerged as a valuable adjunctive technique specifically designed to modify deep coronary calcium. The IVL system uses acoustic pressure waves emitted from a specialized balloon catheter, effectively fracturing both superficial and deep calcium within the arterial wall, thus facilitating more uniform stent expansion and improving procedural outcomes [5,15].

Clinical studies, particularly the Disrupt CAD trials, have consistently demonstrated the safety and efficacy of IVL in facilitating successful PCI in heavily calcified lesions, with favorable procedural and clinical outcomes reported. Compared with rotational atherectomy, IVL carries the advantage of simplicity, lower risk of coronary perforation, reduced vessel trauma, and avoidance of complications related to distal embolization, thus making it a preferred choice for severely calcified lesions [15,16].

However, the use of IVL in the left main coronary artery remains technically demanding and less frequently described in literature. The left main artery's larger diameter, short length, and the critical myocardial territory it supplies pose unique procedural challenges and heightened risks. Limited data exists regarding IVL application specifically in left main lesions, and most available evidence stems from its use in proximal segments of the LAD, right coronary artery (RCA), or circumflex artery [5,17]. Hence, our case illustrates an important contribution to existing literature by highlighting the successful use of IVL to facilitate PCI of a heavily calcified left main lesion in a high-risk patient, underscoring the potential of IVL to expand PCI indications safely into more complex and challenging anatomical territories.

This case underscores several critical lessons in managing high-risk patients with complex coronary artery disease. Foremost is the indispensable role of multidisciplinary Heart Team discussions in guiding therapeutic strategies for complex cases, a practice strongly advocated by contemporary guidelines to optimize patient outcomes [7]. Engaging interventional cardiologists, cardiac surgeons, anesthesiologists, and other relevant specialties ensures comprehensive risk assessment and facilitates individualized patient care.

Equally essential is the concept of shared decision-making, which involves clearly communicating potential benefits and risks to the patient, thereby enabling an informed, patient-centered choice regarding the preferred therapeutic approach. This practice has been increasingly emphasized as fundamental to ethical clinical care and has been shown to improve patient satisfaction, compliance, and clinical outcomes [10].

Furthermore, this case illustrates that innovative and advanced technologies, such as shockwave IVL, can effectively expand treatment options even in anatomically challenging and traditionally high-risk coronary lesions. Careful patient selection, individualized planning, and meticulous procedural execution remain pivotal in achieving optimal outcomes in such scenarios [5].

Our report has some inherent limitations that should be acknowledged. First, the absence of intravascular ultrasound (IVUS) imaging during the procedure represents a notable limitation. IVUS provides detailed characterization of vessel size, lesion morphology, and optimal stent expansion, and its routine use in complex PCI procedures, particularly involving the left main coronary artery, is strongly recommended by contemporary guidelines due to its association with improved clinical outcomes [5,7]. Due to temporary unavailability at our institution, IVUS could not be utilized in this case, which potentially limited precise vessel assessment and optimal stent sizing. Moreover, the absence of IVUS limited our ability to perform detailed vessel assessment and stent optimization, which is particularly recommended in complex left main interventions to improve procedural and long-term outcomes [18].

## Conclusions

This case highlights the feasibility and safety of utilizing shockwave intravascular lithotripsy combined with intra-aortic balloon pump support to successfully treat a heavily calcified left main coronary artery lesion in an elderly high-risk patient deemed unsuitable for surgical revascularization. The role of a multidisciplinary Heart Team discussion and patient-centered shared decision-making was pivotal in guiding the treatment strategy, balancing procedural risks against potential benefits. As innovative technologies such as intravascular lithotripsy continue to advance, they offer promising opportunities to expand the scope of percutaneous coronary intervention into increasingly complex and traditionally surgical territories, particularly when careful patient selection and individualized procedural planning are applied.
